# Antifungal Activity and Potential Mechanism of 6,7, 4′-O-Triacetylscutellarein Combined With Fluconazole Against Drug-Resistant *C. albicans*

**DOI:** 10.3389/fmicb.2021.692693

**Published:** 2021-08-17

**Authors:** Liu-Yan Su, Guang-Hui Ni, Yi-Chuan Liao, Liu-Qing Su, Jun Li, Jia-Sheng Li, Gao-Xiong Rao, Rui-Rui Wang

**Affiliations:** ^1^School of Chinese Materia Medica, Yunnan University of Traditional Chinese Medicine, Kunming, China; ^2^Engineering Laboratory for National Health Theory and Product of Yunnan Province, Yunnan University of Traditional Chinese Medicine, Kunming, China

**Keywords:** *Candida albicans*, drug resistance, 6, 7, 4′-O-triacetylscutellarein, fluconazole, synergistic effect

## Abstract

The increased resistance of *Candida albicans* to conventional antifungal drugs poses a huge challenge to the clinical treatment of this infection. In recent years, combination therapy, a potential treatment method to overcome *C. albicans* resistance, has gained traction. This study assessed the effect of 6,7,4′-O-triacetylscutellarein (TA) combined with fluconazole (FLC) on *C. albicans in vitro* and *in vivo*. TA combined with FLC showed good synergistic antifungal activity against drug-resistant *C. albicans in vitro*, with a partial inhibitory concentration index (FICI) of 0.0188–0.1800. In addition, the time-kill curve confirmed the synergistic effect of TA and FLC. TA combined with FLC showed a strong synergistic inhibitory effect on the biofilm formation of resistant *C. albicans*. The combined antifungal efficacy of TA and FLC was evaluated *in vivo* in a mouse systemic fungal infection model. TA combined with FLC prolonged the survival rate of mice infected with drug-resistant *C. albicans* and reduced tissue invasion. TA combined with FLC also significantly inhibited the yeast-hypha conversion of *C. albicans* and significantly reduced the expression of RAS-cAMP-PKA signaling pathway-related genes (RAS1 and EFG1) and hyphal-related genes (HWP1 and ECE1). Furthermore, the mycelium growth on TA combined with the FLC group recovered after adding exogenous db-cAMP. Collectively, these results show that TA combined with FLC inhibits the formation of hyphae and biofilms through the RAS-cAMP-PKA signaling pathway, resulting in reduced infectivity and resistance of *C. albicans*. Therefore, this study provides a basis for the treatment of drug-resistant *C. albicans* infections.

## Introduction

In recent years, the morbidity and mortality of invasive fungal infections have remained high, especially in patients with weakened immunity and hospitalized patients with severe illness (Noble et al., 2016). *Candida albicans* is the most common opportunistic fungal pathogen, which can cause superficial infections of the skin, oral cavity, and mucous membranes and potentially life-threatening invasive infections (Rajasekharan et al., 2018). Fluconazole has become the first choice for treating *C. albicans* infection due to its good effects, few side effects, and broad antibacterial spectrum. However, long-term, high-dose use of fluconazole can lead to the emergence of drug-resistant strains, which poses a major challenge to the clinical prevention and treatment of *C. albicans* infection (Zhang et al., 2020). Therefore, it is necessary to find new antifungal drugs or effective treatment strategies for inhibiting fungal resistance.

**FIGURE 1:**
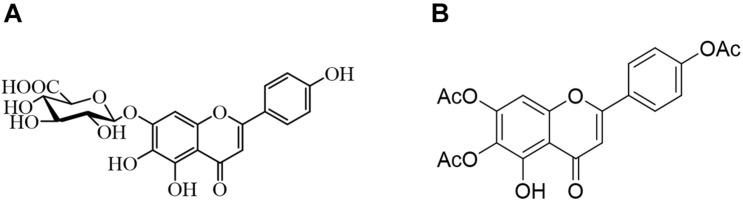
**(B)** Chemical structure of 6,7,4′-O-triacetylscutellarein (TA) and **(A)** scutellarin (SL).

Due to the limited therapeutic effects of existing antifungal drugs, more attempts have been made to identify effective antifungal drugs. Combination therapy has been widely studied and used to combat fungal resistance. Studies have shown that using natural compounds derived from traditional Chinese medicine and their derivatives combined with fluconazole has an excellent synergistic effect against drug-resistant *C. albicans* (Gong et al., 2019). As illustrated in Figure 1, the 6,7,4′-O-triacetylscutellarein (TA) is the structural modification of scutellarin (SL) (Ni et al., 2018). We studied the antifungal activity of TA and SL and unexpectedly found that TA combined with FLC has good antifungal activity against *C. albicans*, especially the resistant strains.

TA is a flavonoid. Previous reports have shown that flavonoids combined with FLC have significant antifungal effects against *C. albicans*, quercetin, baicalein, chalcone, etc. Besides, TA combined with FLC has a good synergistic inhibitory effect on drug-resistant *C. albicans* (Huang et al., 2008; Gao et al., 2016; Wang et al., 2016). Several reports have shown that the antifungal effects of flavonoids and their derivatives are related to the inhibition of biofilm formation, yeast-hyphae transition, and efflux pump activity. However, the anti-*C. albicans* activity of TA has not been explored. Therefore, this study investigated whether TA combined with FLC has antifungal activity against *C. albicans* and whether they can treat drug resistance *C. albicans* infection.

Herein, the antifungal activity of TA combined with FLC was determined both *in vitro* and *in vivo*. In addition, this study also investigated whether TA combined with FLC can inhibit yeast-hyphae transformation through the RAS-cAMP-PKA signaling pathway to combat drug-resistant *C. albicans*.

## Materials and Methods

### Compounds, Strains, and Culture Conditions

We prepared a TA and SL solution (25 mg/mL) in DMSO (dimethyl sulfoxide). Fluconazole (FLC) was obtained from Helioeast company (Nanchang, China) and dissolved in DMSO (10 mg/mL). All drug solutions were stored at 4°C.

Professor Li Yu-ye of the First Affiliated Hospital of Kunming Medical University of China donated clinically resistant *C. albicans* strains including CA3511, CA602, A550, CA4508, CA381, CA187, CA800, CA799, CA3816, CA808, and CA23. In addition, the standard *C. albicans* strain SC5314 was sourced from Yunnan Denglou Technology Co., Ltd. All the strains were frozen with 30% glycerol in a refrigerator at −80°C. Before the experiment, the strains were incubated in an incubator at 37°C on Sabouraud-dextrose-agar (SDA) for at least 24 h.

### Antifungal Susceptibility Testing

According to the Clinical and Laboratory Standards Institute (CLSI) M27-A3 method (Reyes and Ghannoum, 2000), a broth microdilution method was used to determine the minimum inhibitory concentration (MIC) of samples used alone and in combination with FLC against *C. albicans* strains. Here MIC is defined as the drug concentration that reduces growth by 80% compared to the control group. Serial five-fold dilutions of the samples were prepared using Sabouraud-dextrose-broth (SDB) medium (Weerasekera et al., 2016), with a final concentration of 0.32–1000 μg/mL. These were used in a preliminary study on the antifungal activity of TA and SL. Serial two-fold dilutions of the samples were then prepared using SDB to further analyze the antifungal activity of TA combined with FLC. The final concentration of the samples was 32–1000 μg/mL when used alone and 0.064–200 μg/mL and 0.0128–40 μg/mL when used in combination (TA and FLC, respectively). Each well contained 100 μL of 1 × 10^5^ colony-forming units (CFU)/mL of yeast cell suspension. Then, we added 50 μL of TA and FLC to each well (200 μL). Subsequently, we incubated the flat bottom 96-well plate (701001, NEST, China) at 37°C for 24 h. The wells with only fungi (no drugs) were defined as the control and wells with only SDB as the blank. Further, we determined the growth rate in each well via visual observation and measuring the optical density at a wavelength of 625 nm using a multifunctional microplate reader (SpectraMax 340PC384, Molecular Devices Corporation, United States). We analyzed the antifungal effects using fractional inhibitory concentration index (FICI) to assess the *in vitro* interactions between TA and FLC. The FICI was calculated for each combination using the following formula: FICI = FICI TA + FICI FLC = (MIC of TA in combination/MIC of TA alone) + (MIC of FLC in combination/MIC of FLC alone). Here, synergy was defined as FICI ≤ 0.5. 0.5 < FICI < 1 indicated partial synergy, FICI = 1 indicated additive effect, 1 < FICI ≤ 4 indicated indifference, and antagonism was defined as FICI > 4 (Khodavandi et al., 2010).

### Antifungal Kinetics Assay

The optical density (OD) of different drug treatment groups was measured at different time points. The time-course curve of fungal killing was plotted to study the dynamic inhibitory effect of TA on drug-resistant *C. albicans* CA23. Next, we diluted the overnight cultures of *C. albicans* strains with SDB medium to 1 × 10^5^ CFU/mL and exposed them to TA (128 μg/mL), FLC (16 μg/mL), and a combination of TA (128 μg/mL) with FLC (16 μg/mL). It is worth noting that in the control group, only fungi were not added with drugs. Subsequently, we incubated the cells at 37°C with constant shaking (150 rpm), and 200 μL samples collected from each well after 0, 4, 8, 12, 24, 48, and 72 h to measure the OD at 625 nm (Li X. et al., 2020; Li Y. et al., 2020; Li et al., 2021). The experiment was performed thrice (independently), and each sample was assessed in three replicates.

### The Effect of FLC Combined With TA on the Pre-formation and Formation of *C. albicans* Biofilm

We evaluated the effect of TA on the formation and pre-formation of the biofilm of drug-resistant *C. albicans* CA23 through microscopic observation and cell staining (Soll and Daniels, 2016; Wu et al., 2020). First, we cultured *C. albicans* CA23 in SDB medium overnight, then washed the fungal cells three times with PBS, resuspended them in RPMI-1640 (+10% FBS) medium, and adjusted it to 1 × 10^5^ CFU/mL (final concentration). Next, we added 1 mL of the fungal cell suspension of different groups to the 24-well plate; statically incubated for 90 min at 37°C for adhesion; aspirated to remove non-adherent cells; then added the same volume of Fresh RPMI 1640 (+10% FBS) medium. The plate was incubated in a 37°C incubator for 24 h until a mature biofilm was formed. After 90 min of adhesion, we added fresh RPMI 1640 (+10% FBS) containing the drug to the 24-well plate, and the plate was incubated at 37°C for 24 h to detect the formation of biofilm by TA impact. In addition, after 90 min of adhesion, we added fresh RPMI 1640 (+10% FBS) to the 24-well plate, incubated the plate at 37°C for 24 h to form a mature biofilm then discarded the biofilm supernatant. Fresh RPMI 1640 medium (+10% FBS) containing the drug was then added. The incubation continued for 24 h to detect the effect of TA on pre-formation biofilm. Lastly, the plates were incubated at 37°C for 24 h to observe the antibiofilm effect of TA. We divided them into four groups: control, FLC, TA, and TA + FLC. Each experiment had three multiple holes per group.

### Evaluation of Antifungal Activity *in vivo* Using Mouse Systemic Fungal Infection Model

#### Experimental Design and Mouse Model

We evaluated the antifungal activity of TA in a murine model of systemic infection *in vivo* (Kong et al., 2018; Khan et al., 2020). We raised and treated all mice following the guidelines approved by the China Animal Protection and Use Committee. A model of systemic fungal infection was established in 60 male and female (1:1) C57BL/6 mice (aged 6–8 weeks, weighing 18–22 g). After 1 week of adaptive feeding, Subsequently, the mice were randomly assigned to the following experimental groups: Blank (without cyclophosphamide and *C. albicans*), Control (without *C. albicans*), Model (only *C. albicans*), FLC (2 mg/kg), TA (200 mg/mL), and TA (200 mg/mL) + FLC (2 mg/kg). we intraperitoneally injected the mice with cyclophosphamide (100 mg/kg body weight) for three consecutive days to induce an immunodeficiency model. On the 4th day, we injected the drug-resistant *C. albicans* suspension (3.75 × 10^6^ CFU/mL, 0.1 mL/10 g) through the tail vein to cause systemic infection in the mice. Two hours after model establishment, the experimental mice were dosed intragastrically by weight. Notably, the blank, control, and model mice groups were prepared using a carboxymethylcellulose sodium (CMC-Na) gelling agent.

#### Mice Weight and Survival Assay

The general state of the experimental animals, including activity status, hair status, weight, and survival rate changes, was observed after successful treatment with *C. albicans*. In addition, the survival status of mice in each group was monitored and recorded for 14 days.

#### Determination of Fungal Burden

The mice were sacrificed via cervical dislocation under complete anesthesia on the 14th day, and kidneys were collected and weighed. Next, an appropriate amount of PBS (10 μL sterile PBS was needed for 1 mg of internal organs) was added and ground with a homogenizer. Finally, 10 μL of the tissue homogenate was placed on the SDA plate and incubated at 37°C for 48 h to determine the fungal burden in the kidneys. The experiments were performed thrice (independently). The results were expressed as log10^CFU/g^, CFU/g = (Number of CFU × dilution times)/weight of tissue.

#### Histopathological Study of Mouse Kidney

The kidney was dissected and placed in 10% buffered neutral formalin for 24 h. The fixed and shaped tissue was embedded in paraffin, sectioned at a thickness of 5 μm, deparaffinized, and dehydrated using standard techniques. The sections were stained with hematoxylin and eosin (HE) and periodic acid methylamine silver (PASM) to detect inflammatory cells and hyphae. The field of view was randomly selected to observe histopathological changes using an optical microscope at a certain magnification.

### Filamentation Assay in a Liquid Medium

To evaluate the effect of TA on the filamentous growth of *C. albicans*, we used Spider medium (1% mannitol, 1% nutrient broth, 0.2% K_2_HPO_4_, pH 7.2), and Sabouraud Dextrose medium (10 g peptone and 40 g dextrose in 1000 mL ddH2O) supplemented with 10% FBS for hyphae induction experiment (Yang et al., 2018). Four groups were set for this assay: TA (128 μg/mL), FLC (16 μg/mL), a combination of TA (128 μg/mL) with FLC (16 μg/mL), and control group (no drug added). Drugs and *C. albicans* cells (1 × 10^5^ CFU/mL) were added to the hyphae induction medium and incubate at 37°C for 4 h. An inverted microscope (Carl Zeiss) with a camera was used to visualize and take images. The experiment was performed thrice (independently), and each sample was evaluated in three replicates.

### Quantitative Real-Time PCR Assays

Four groups were set for this assay: Control, TA (128 μg/mL), FLC (16 μg/mL), and a combination of TA (128 μg/mL) with FLC (16 μg/mL). Planktonic cells of *C. albicans* treated with the drug and grown in RPMI-1640 (+10% FBS) medium at 37°C for 16 h were diluted to 1 × 10^5^CFU/mL cell density. The fungal cells were washed three times with sterile PBS and harvested by centrifugation at 3900 rpm for 5 min. Then, the fungal cells were ground into a powder with liquid nitrogen. TRIzol (Invitrogen, United States) was used to extract total RNA according to the instructions. The quality and quantity of the extracted RNA were determined spectrophotometrically (NanoDrop Lite, United States). The Reverse Transcription System kit (Promega, United States) was used to convert the total RNA (1 μg) into cDNA with random primers in a 20 μL reaction volume following the manufacturer’s instructions. The primer sequences used for amplification of specific genes are shown in Table 1. RT-PCR mixtures contained; 2 μL cDNA, 10 μL GoTaq^®^ qPCR master mix (Promega, United States), 0.5 μL of each primer at a concentration of 10P, and sterile Nuclease-Free water to a final volume of 20 μL. The lightcycler 96 fluorescent quantitative PCR system was used for qRT-PCR analysis. The cycles were as follows: pre-denaturation at 95°C for 10 min, then 95°C for 15 s, 55°C for 30 s, and 72°C for 30 s (40 cycles). Actin (ACT1) was used as the internal control. The transcription level of the selected genes was calculated using the 2^–ΔΔCt^ method. Three independent experiments were performed, each in triplicate.

**TABLE 1:** Primers used in this study.

Oligo name	Sequence (5′ to 3′)	Product length (bp)
*ACT1*-F	ACGGTGAAGAAGTTGCTGCT	180
*ACT1*-R	TGGATTGGGCTTCATCACCA	
*RAS1*-F	GTGGTGGTGTTGGTAAATCCG	178
*RAS1*-R	TGTTCTCTCATGGCCAGATATTC	
*EFG1*-F	AATGTGGCCCAAATGACACG	131
*EFG1-*R	TTGGCAACAGTGCTAGCTGA	
*ECE1*-F	TGCCTGTGCTACTGTTTTTGC	123
*ECE1*-R	ACAGTAGGTGCTTGGTCAGC	
*HWP1*-F	ACTGAACCTTCCCCAGTTGC	185
*HWP1*-R	GTCGTAGAGACGACAGCACT	

### cAMP Rescue Assay

We prepared *C. albicans* cells in RPMI-1640 (1 × 10^5^ CFU/mL) supplemented with 10% FBS to verify the role of TA combined with FLC in inhibiting the Ras1-cAMP-Efg1 pathway. We added dibutyryl-cAMP (db-cAMP) (MCE, China) immediately after adding the drug to make the final concentration to 128 μM. Cells not treated with db-cAMP were used as controls. An inverted microscope (Carl Zeiss) with a camera was used to observe and take images after incubation at 37°C for 4 h. Each sample was assessed in three replicates.

### Statistical Analysis

GraphPad Prism 8 was used for all statistical analyses. The data are expressed as the average of three repeated experiments ± standard deviation (SD). The differences among the groups were evaluated using analysis of variance (ANOVA). *P* < 0.05 was considered statistically significant (^∗^*p* < 0.05, ^∗∗^*p* < 0.01, ^∗∗∗^*p* < 0.001, ^****^p < 0.0001).

## Results

### Antifungal Susceptibility Testing

TA or SL alone had no antifungal effect on sensitive and resistant *C. albicans* (MIC > 1000 μg/mL) (Table 2). However, SL combined with FLC showed antagonistic and additive effects on sensitive and resistant strains (FICI, 14.55 and 1.00, respectively). In contrast, TA combined *with* FLC showed an indifference effect and a strong synergistic effect on sensitive and resistant strains (FICI, 2.27 and 0.18, respectively). We used ten clinically drug-resistant *C. albicans* for further antifungal activity experiments to verify whether TA has the same antibacterial effect on all drug-resistant *C. albicans*. TA had no antifungal effect on all drug-resistant *C. albicans* when used alone (MIC > 1000 μg/mL) but had strong synergy on all drug-resistant *C. albicans* when combined with FLC (FICI < 0.5) (Table 3).

**TABLE 2:** Antifungal activity of TA and SL against *C. albicans* (*n* = 3).

Drugs	MIC (μg/ml)		FICI		Interaction	
			
	SC5314	CA3511	SC5314	CA3511	SC5314	CA3511
FLC	6.50	>1000	–	–	–	–
SL	>1000	>1000	–	–	–	–
FLC + SL	94.30	>1000	14.55	1.00	ANT	ADD
TA	>1000	>1000	–	–	–	–
FLC + TA	14.70	180.06	2.27	0.18	IND	SYN

*CA, *C. albicans*; SL, scutellarin; TA, 6,7,4′-O-triacetylscutellarein; FLC, fluconazole; MIC denotes the minimum inhibitory concentration of drug that inhibited fungal growth by 80% compared with the growth control; FICI, fractional inhibitory concentration index; ANT, antagonistic; ADD, additive; IND, indifferent; SYN, synergism. MIC values and FICIs are shown as the median of three independent experiments.*

**TABLE 3:** The effect of FLC combined with TA against *C. albicans in vitro* (*n* = 3).

Strains	MIC (μg/ml)				FICI	Interaction
	
	Alone		Combined			
		
	FLC	TA	FLC	TA		
CA602	105.11	>1000	2.61	13.04	0.0313	SYN
CA550	127.56	>1000	5.89	29.45	0.0609	SYN
CA4508	870.45	>1000	20.24	101.21	0.0738	SYN
CA381	>1000	>1000	10.05	50.28	0.0301	SYN
CA187	>1000	>1000	6.27	31.36	0.0188	SYN
CA800	>1000	>1000	12.15	60.75	0.0364	SYN
CA799	>1000	>1000	14.91	74.59	0.0447	SYN
CA3816	>1000	>1000	13.19	65.96	0.0395	SYN
CA808	>1000	>1000	7.81	39.06	0.0234	SYN
CA23	>1000	>1000	24.61	123.06	0.0738	SYN

*CA, *C. albicans*; TA, 6,7,4′-O-triacetylscutellarein; FLC, fluconazole; MIC denotes the minimum inhibitory concentration of drug that inhibited fungal growth by 80% compared with the growth control; FICI, fractional inhibitory concentration index; SYN, synergism. MIC values and FICIs are shown as the median of three independent experiments.*

### Time-Kill Curve

**FIGURE 2:**
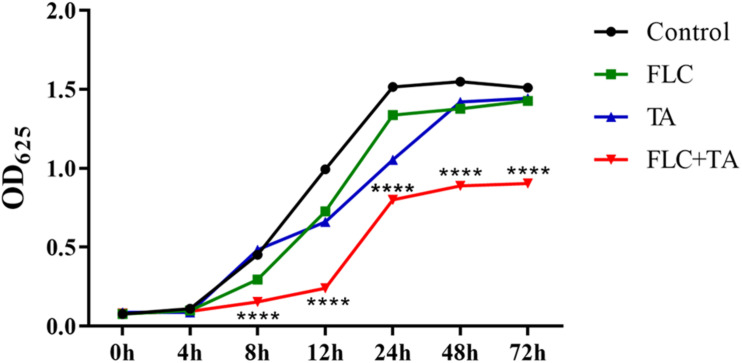
Time-killing curves of *C. albicans* CA23 treated with TA and TA combined with FLC. The combination group was compared with the FLC group. ****P < 0.0001.

TA and FLC showed no antifungal effect on drug-resistant *C. albicans* CA23 when used alone (Figure 2). In contrast, combined TA and FLC showed strong synergistic antifungal activity after 8 h of treatment, which was very significant even at 72 h (^****^P < 0.0001).

### The Effect of FLC Combined With TA on *C. albicans* Biofilm Pre-formation and Formation

**FIGURE 3:**
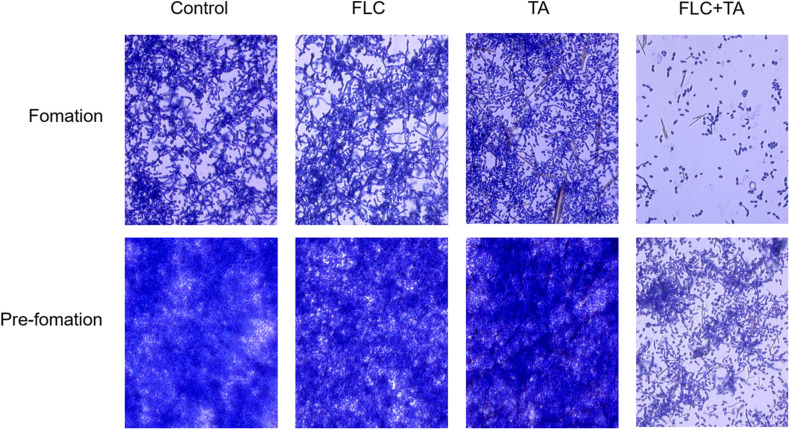
The effect of TA combined with FLC on *C. albicans* biofilm. The morphology of biofilm and hyphae were observed using a microscope (magnification 400×) after incubation at 37°C for 24 h and staining with 0.4% crystal violet. FLC combined with TA effectively inhibited *C. albicans* biofilm formation and positively affected the pre-formed *C. albicans* biofilm.

The *C. albicans* in the control group formed mature biofilms after 24 h of culture (Figure 3). The biofilm formation of the FLC and TA groups was not significantly inhibited compared with that of the control group, forming a nearly mature biofilm with yeast, hyphae, and pseudohyphae. However, biofilm formation in the TA and FLC combined group was strongly inhibited. The fungal cells were sparsely distributed in the FLC + TA group, with most being in the yeast state. For pre-formed biofilm analysis, we first cultivated *C. albicans* for 24 h to form a mature biofilm, then added drugs for further cultivation for 24 h. The FLC and TA groups did not significantly prevent the further growth and development of mature biofilms compared with the control group. However, TA combined with FLC destroyed the structure of mature biofilms, inhibiting further development of mature biofilms.

### Evaluation of Antifungal Activity *in vivo* Using Mouse Systemic Fungal Infection Model

#### Mice Weight and Survival Assay

**FIGURE 4:**
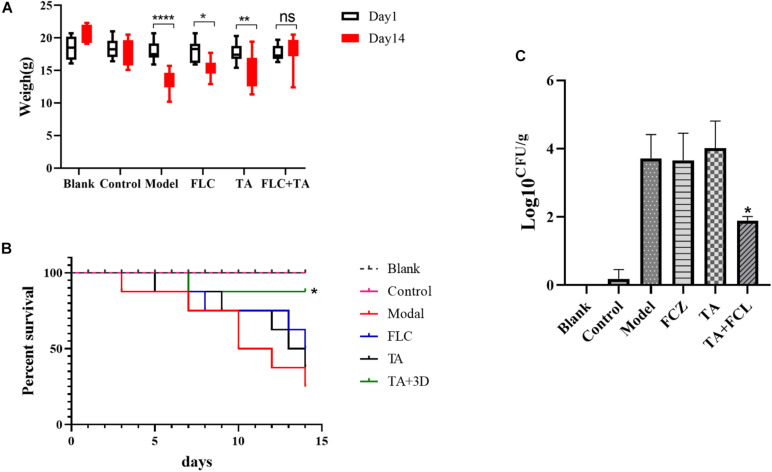
The effect of FLC combined with TA on the infection of drug-resistant *C. albicans in vivo*. **(A)** The effect of FLC combined with TA on the mice body weight. The mice in the model group, TA, and FLC groups significantly lost weight compared with the weight at the first and last days of administration. In contrast, the body weight of the mice in the FLC and TA combination group was not significantly changed. **(B)** The effect of FLC combined with TA on the survival rate of mice. The survival rate of mice was higher in the combined administration group than in the model group. **(C)** Kidney fungal burden. The number of kidney fungi in mice significantly reduced in the TA combined with the FLC group after 14 days of treatment compared with the model group. *P < 0.05, **P < 0.01, ****P < 0.0001.

We first performed weight changes and survival analyses on mice infected with drug-resistant *C. albicans* (CA23) to preliminarily evaluate the antibacterial efficacy of different drug groups *in vivo*. The body weight of mice in the model group, FLC group, and TA group significantly decreased compared with the body weight at first and last days of administration (Figure 4A). However, the weight of the mice in the TA combined with the FLC group was not significantly altered (*P* > 0.05). Similarly, there was no significant difference in the survival rate of the model group, FLC group, and TA group (*P* > 0.05) after 14 days of the administration (the survival rates; 25, 40, and 40%, respectively) (Figure 4B). However, TA combined with FLC significantly increased the survival rate of mice to 85% (*P* < 0.05). Collectively, these findings indicate that the combination of TA and FLC can significantly improve the survival rate of mice infected with drug-resistant *C. albicans* compared with FLC monotherapy, confirming their antifungal activity in the body.

#### Determination of Fungal Burden

We conducted a fungal burden analysis to assess the effect of TA combined with FLC on the amount of fungal colonization in the kidney of mice infected with drug-resistant *C. albicans* systemic infection. The fungal burden both the FLC and TA groups was not significantly different compared with the model group (*P* > 0.05) (Figure 4C). However, the fungal kidney burden in the TA + FLC group was significantly reduced compared with the control, FLC, and TA groups (*P* < 0.01).

#### Histopathological Study of Mouse Kidney

**FIGURE 5:**
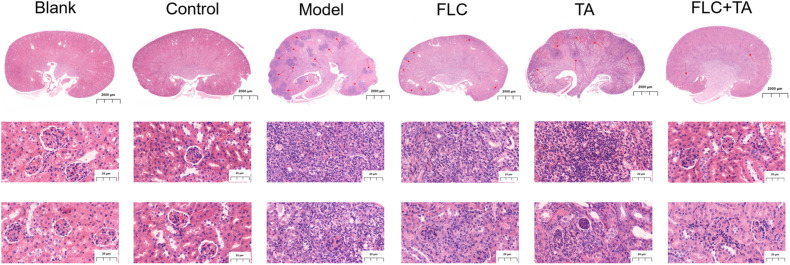
A representative H&E stained section of the kidney. The infiltration of inflammatory cells in the kidney of mice in the combined administration group was significantly reduced. The picture of the whole kidney field is enlarged by 80×, and the other images are enlarged by 400×. The red arrow refers to the characteristic area of inflammatory cell infiltration in mouse kidney tissue.

**FIGURE 6:**
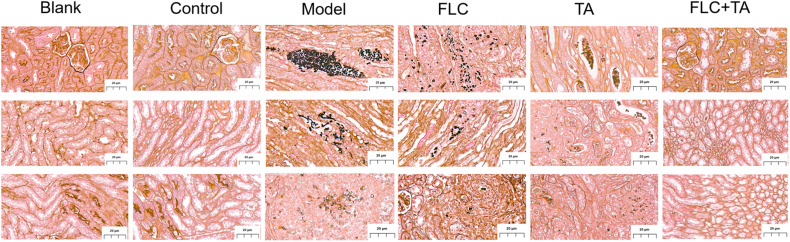
Representative PASM stained section of the kidney. The fungal burden in the kidneys of mice in the combined administration group was significantly reduced (magnification of 400×).

The mouse kidney sections were stained with HE and PASM to further assess the fungal burden of mouse kidneys. The HE staining results (Figure 5) showed that the kidneys of the Blank and Control group mice did not have inflammatory cell infiltration. However, there was a significant inflammatory cell infiltration in the kidneys of mice in the model and TA and FLC group. In contrast, the infiltration of inflammatory cells in the kidney of mice in TA combined with the FLC group was greatly reduced and was close to that of normal mice. Furthermore, the fungi were stained black or gray-brown in the PASM-stained kidneys (Figure 6). Fungi did not invade the kidneys of the mice in the blank and the control groups. However, several fungi invaded the kidneys of mice in model, TA, and FLC groups. Besides, most of these fungi were hyphae. In contrast, there were very few yeast-like fungi in the kidneys of mice in the TA combined with the FLC group.

### Filamentation Assay in a Liquid Medium

**FIGURE 7:**
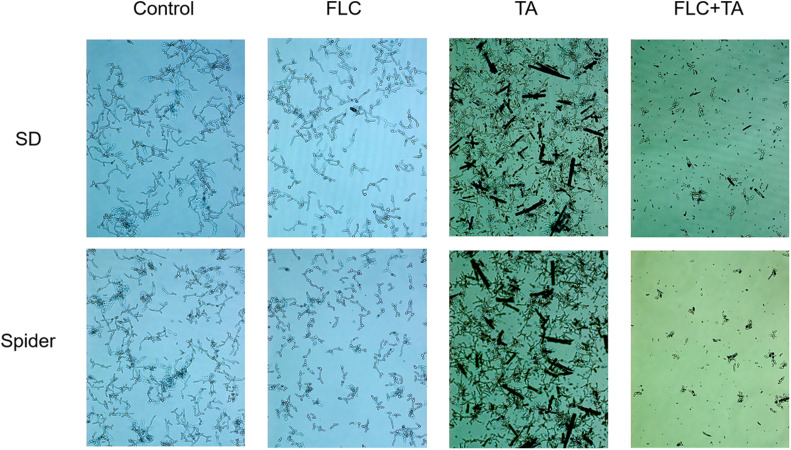
Filamentation assay in a liquid medium. The SD and Spider mycelium induction medium significantly inhibited the growth of drug-resistant *C. albicans* CA23 hyphae in the combined administration group than in the control group (magnification; 400×).

The pathogenic state of *C. albicans*, hyphae, plays a vital role in the pathogenesis of *C. albicans* (Chen et al., 2020; Li X. et al., 2020; Li Y. et al., 2020). Spiders and Synthetic Dropout (SD) media were used to induce hyphae formation. *C. albicans* CA23 formed dense and long hyphae in the Spider and SD of the control group (Figure 7). In addition, there was no significant difference in the mycelial growth state between the TA and FLC disposable groups and the control group. On the contrary, the length of the hyphae was shorter in the TA combined FLC group than in the other groups. The number of hyphae was also significantly reduced in the TA combined FLC group than in the other groups.

### Quantitative Real-Time PCR Assays

**FIGURE 8:**
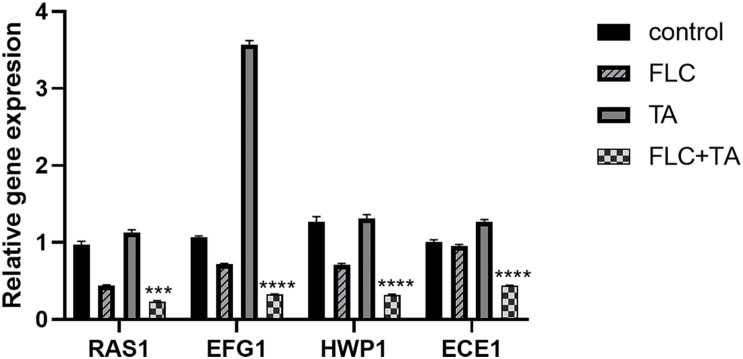
The mRNA transcription levels of the Ras-cAMP-PKA pathway and hyphae-related genes. The mRNA transcripts were significantly down-regulated in the TA combined with the FLC group. ACT1 was used as an internal reference gene and quantified using the 2^– ΔΔCT^ method. ***p < 0.001, ****p < 0.0001.

We also conducted RT-PCR experiments on the RAS-cAMP-PKA pathway-related genes RAS1 and EFG1 and the hypha-related genes (HWP1 and ECE1) to further assess the antifungal mechanism of TA combined with FLC. RAS1, EFG1, HWP1, and ECE1 expressions were significantly down-regulated in the FLC group and up-regulated in the TA group compared with the control group (Figure 8). Interestingly, the following genes were significantly downregulated in the TA combined FLC group compared with the FLC group: RAS1 > 2 times, EGF1 > 2.3 times, HWP1 > 2.25 times, and ECE1 > 2.20 times.

### cAMP Rescue Assay

**FIGURE 9:**
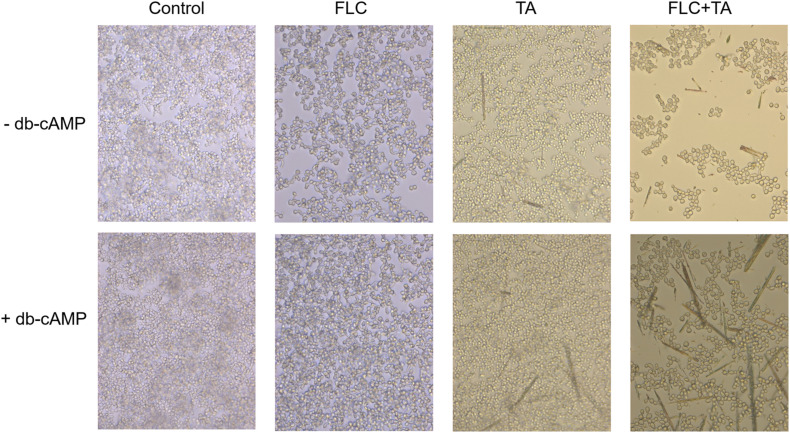
cAMP is related to the formation of hyphae and biofilm of *C. albicans*. The exogenous cAMP restored the mycelium formation of *C. albicans* inhibited by TA combined with FLC (magnification of 400×).

Cyclic adenosine monophosphate (cAMP) level is essential for activating the RAS-cAMP-PKA signaling pathway (Huang et al., 2019). We confirmed that cAMP is involved in the transition from yeast to hyphae by adding db-cAMP to the medium. The db-cAMP addition successfully rescued the inhibitory effect of TA combined with FLC on the formation of hyphae, partially restoring the ability of *C. albicans* to form hyphae (Figure 9). Therefore, the combination of TA and FLC can reduce the cAMP level in fungal cells.

## Discussion

Several studies have focused on discovering new antifungal drugs and finding new treatment strategies to solve the increasing fungal drug resistance. Herein, TA combined with FLC showed a better synergistic effect against drug-resistant *C. albicans* than SL. A previous experiment showed that TA combined with FLC has an indifference and a strong synergistic effect on sensitive and resistant *C. albicans* (FICI; 2.27 and 0.18, respectively). Therefore, we used ten clinically drug-resistant *C. albicans* for further antifungal activity experiments to verify whether TA has the same antifungal effect on all drug-resistant *C. albicans*. TA combined with FLC showed a strong synergistic effect on all drug-resistant *C. albicans* (FICI < 0.5). Besides, the MIC of FLC decreased from 105.11–1000 μg/mL to 2.61–24.61 μg/mL. We also used the time-kill curve to confirm the synergistic effect of TA combined with FLC.

Persistent *C. albicans* infections in the hospital are often closely related to biofilm formation (Cavalheiro and Teixeira, 2018). Biofilms can reduce the permeability of antifungal drugs and are inherently resistant to antibacterial drugs. Reports have shown that the resistance of *C. albicans* biofilms can reach 1000 times that of planktonic *C. albicans* (Tsui et al., 2016). Therefore, inhibiting biofilm formation can clinically inhibit resistant *C. albicans* infection. Herein, the drug-resistant *C. albicans* CA23 formed a mature biofilm after 24 h of culture (Gulati and Nobile, 2016). These results show that the combination of TA + FLC interacts with fungi for 24 h, making most fungi remain in the yeast state, thus significantly inhibiting the CA23 biofilm formation. The combination of TA + FLC also destroyed the structure of the pre-formed biofilm, inhibiting its further development. Therefore, TA combined with FLC could be a promising strategy for treating biofilm-related infections caused by drug-resistant *C. albicans.*

However, *in vivo* experiments were needed to prove the safety and effectiveness of the drug. Herein, we established a mouse model of systemic infection using drug-resistant *C. albicans.* The study showed that the weight of the mice in the TA combined with the FLC treatment group recovered, and the survival rate was significantly improved compared with the weight and survival rate of mice in the FLC group (*P* < 0.05). In addition, a comprehensive analysis of the kidney, the main target organ of invasive *C. albicans, i*nfection was also conducted (Chen et al., 2018). The results showed that the TA combined with the FLC treatment group had a significantly lower kidney fungal burden (*P* < 0.05), reduced number of lesions, and inflammatory cell infiltration. Besides, they compared with the TA and FLC groups, the fungal cells were significantly reduced, especially the hyphae cells. In summary, the synergistic antibacterial effect of TA combined with FLC could be due to the inhibition of *C. albicans* yeast-hyphae transformation.

*Candida albicans* has a distinct biological feature since it can grow as yeast, pseudohyphae, and hyphae (Sudbery, 2011). However, the hyphae form has been described as a potential virulence factor of *C. albicans*, which induces pathogenicity by invading epithelial cells, causing tissue damage (Meng et al., 2019). In addition, as an important member of biofilm, hyphae is essential for the formation and stability of biofilm (Desai and Mitchell, 2015). Therefore, it is necessary to explore whether drugs inhibit morphological transformation. Herein, the hyphae of the TA and FLC groups were long and dense after 4 h of incubation in hyphae induction medium, while the hyphae of the TA combined with the FLC group were short and sparse, and most of the remaining bacterial cells remained Yeast form, consistent with the above *in vivo* and *in vitro* results. Therefore, the synergistic effect of TA combined with FLC on drug-resistant *C. albicans* and its biofilm could be due to the inhibitory activity on hyphae.

The EFG1 acts as a positive regulator of yeast-hyphae morphogenesis and mainly depends on the RAS-cAMP-PAK signaling pathway (Sun et al., 2019). The RAS-cAMP-PAK signaling pathway produces the required cAMP-activated protein kinase (PKA). Real-time RT-PCR analysis was used to assess further the antifungal mechanism of TA combined with FLC. We analyzed the expression of Ras/cAMP/PKA signaling pathway-related genes (RAS1 and EFG1) (Lee et al., 2013) and hypha-related genes (HWP1 and ECE1) (Sharkey et al., 1999). Ras1 is a signal transduction GTPase that activates the Ras/cAMP/PKA pathway in *C. albicans* (Li et al., 2014). On the other hand, ECE1 induces cell adhesion and hypha formation by regulating the degree of cell elongation (Engku Nasrullah Satiman et al., 2020). HWP1 is a unique adhesion gene expressed on the surface of mycelium, encoding the cell wall protein of *C. albicans.* Biofilms without the HWP1 gene have weak adhesion to objects (Yan et al., 2019). RAS1, EFG1, HWP1, and ECE1 expressions were significantly down-regulated in the TA combined with the FLC group (RAS1 > 2 times, EGF1 > 2.3 times, HWP1 > 2.25 times, and ECE1 > 2.20 times).

**FIGURE 10:**
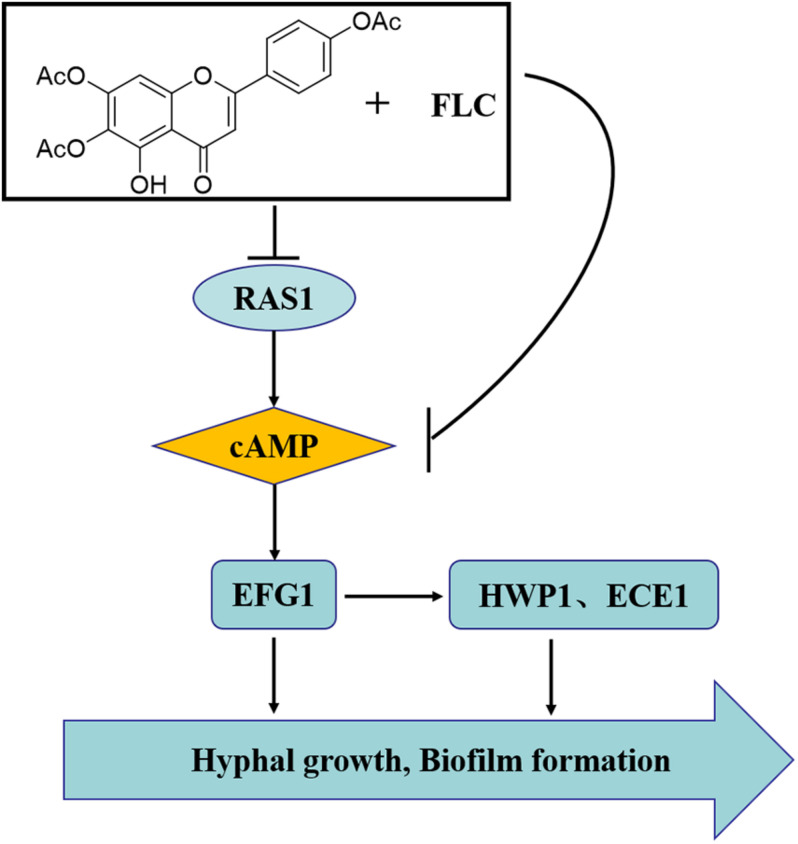
The synergistic mechanism of TA combined with FLC against *C. albicans*. The synergistic mechanism may be related to the changes of downstream hyphae-related genes caused by the RAS-cAMP-PKA pathway inhibition.

We also added db-cAMP, a functional analog of cAMP, to evaluate whether *C. albicans* treated with TA combined with FLC can restore mycelial growth function in culture for further verification. db-cAMP partially restored the mycelium-forming ability o*f C. albicans*, consistent with previous studies, which showed that TA combined with FLC mainly exerts a synergistic anti-*C. albicans* effect by inhibiting the Ras1-cAMP-PKA pathway (Figure 10).

## Conclusion

In conclusion, we report for the first time that TA combined with FLC has a good synergistic antifungal effect on azole-resistant *C. albicans* both *in vitro* and *in vivo*. Furthermore, our mechanism studies have shown that the morphological transformation inhibition causes the synergy via the RAS-cAMP-PKA signaling pathway. Therefore, there is a need to explore more synergy mechanisms in the future.

## Data Availability Statement

The original contributions presented in the study are included in the article/Supplementary Material, further inquiries can be directed to the corresponding authors.

## Ethics Statement

All animals were maintained and treated per the guidelines approved by the Animal Care and Use Committee of China. This study was approved by the Animal Care and Welfare Committee of Yunnan University of Traditional Chinese Medicine (R-0620160027).

## Author Contributions

G-XR and R-RW: providing an overall idea of the experiment. L-YS and G-HN: experimental design and article writing. Y-CL and L-QS: data collation and analysis. JL and J-SL: animal experiment. All authors contributed to the article and approved the submitted version.

## Conflict of Interest

The authors declare that the research was conducted in the absence of any commercial or financial relationships that could be construed as a potential conflict of interest.

## Publisher’s Note

All claims expressed in this article are solely those of the authors and do not necessarily represent those of their affiliated organizations, or those of the publisher, the editors and the reviewers. Any product that may be evaluated in this article, or claim that may be made by its manufacturer, is not guaranteed or endorsed by the publisher.
