# Supplementary figures and images for: Antifungal Activity and Potential Mechanism of 6,7, 4′-O-Triacetylscutellarein Combined With Fluconazole Against Drug-Resistant C. albicans
Source: Front Microbiol. 2021 Aug 17;12:692693. doi: 10.3389/fmicb.2021.692693 (PMC8415886; doi:10.3389/fmicb.2021.692693)

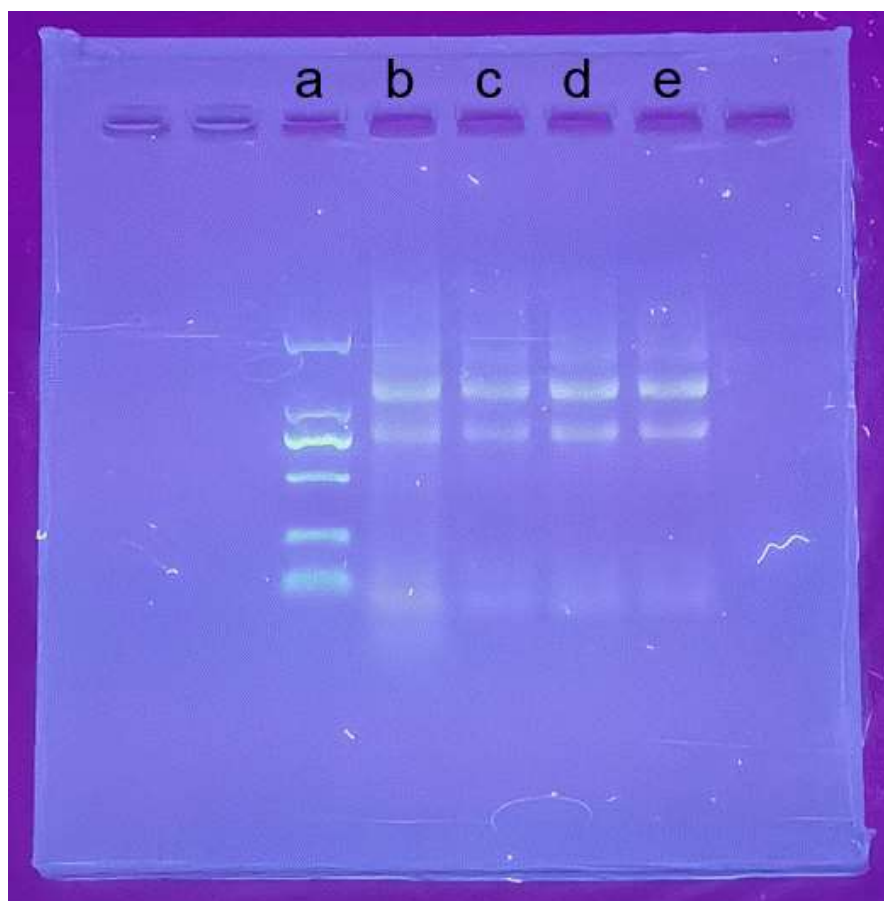

a, Maker; b, TA+FLC group; c, TA group;  
d, FLC group; e, Control group.

Supplement: Supplementary file 1 [file Image_1.pdf]
